# Draft Genome Sequence of *Pseudomonas putida* Strain GM4FR, an Endophytic Bacterium Isolated from *Festuca rubra* L.

**DOI:** 10.1128/genomeA.00086-17

**Published:** 2017-03-30

**Authors:** Franziska Wemheuer, Jacqueline Hollensteiner, Anja Poehlein, Sandra Granzow, Rolf Daniel, Stefan Vidal, Bernd Wemheuer

**Affiliations:** a Agricultural Entomology, Department of Crop Sciences, Georg-August University Göttingen, Göttingen, Germany; b Genomic and Applied Microbiology & Göttingen Genomics Laboratory, Institute of Microbiology and Genetics, Georg-August University Göttingen, Göttingen, Germany

## Abstract

*Pseudomonas putida* GM4FR is an endophytic bacterium isolated from aerial plant tissues of *Festuca rubra* L. Functional annotation of the draft genome (7.1 Mb) revealed 6,272 predicted protein-encoding genes. The genome provides insights into the biocontrol and plant growth-promoting potential of *P. putida* GM4FR.

## GENOME ANNOUNCEMENT

Beneficial plant-associated bacteria promote plant growth and health using a variety of mechanisms, including the production of phytohormones (1, 2). These bacteria can enhance the resistance of their host plant against biotic and abiotic stressors (2). Several members of the genus *Pseudomonas* are known as plant growth-promoting bacteria (2, 3). These include *P. putida* strains, which have been shown to act as efficient biocontrol agents against phytopathogens and nematodes (3, 4).

Here, we report the draft genome sequence of the endophyte *P. putida* GM4FR. This strain was isolated from surface-sterilized aerial tissues of healthy *Festuca rubra* L. plants. Samples were collected from the GrassMan experimental field (5). Genomic DNA of *P. putida* GM4FR was extracted using the MasterPure complete DNA purification kit (Epicentre, Madison, WI, USA). Obtained DNA was used to generate Illumina paired-end sequencing libraries. Sequencing was performed by employing a MiSeq system and the MiSeq reagent kit version 3 (600 cycles) as recommended by the manufacturer (Illumina, San Diego, CA, USA). Quality filtering using Trimmomatic version 0.32 (6) resulted in 5,419,862 paired-end reads. *De novo* genome assembly was performed with the SPAdes genome assembler version 3.8.0 (7). The assembly resulted in 79 contigs (>500 bp) and an average coverage of 144-fold. The assembly was validated and the read coverage determined with QualiMap version 2.1 (8).

The draft genome of *P. putida* strain GM4FR consists of 7,064,252 bp with an overall G+C content of 63.45%. Gene prediction and annotation were performed using Rapid Prokaryotic Genome Annotation (Prokka) (9). The draft genome harbored 10 rRNA genes, 55 tRNA genes, 2,867 protein-encoding genes with functional prediction, and 3,405 genes coding for hypothetical proteins. For phylogenetic classification of *P. putida* GM4FR, multilocus-sequence typing was performed according to Gomila et al. (10). The closest relative of the *P. putida* strain GM4FR is *P. putida* KT2440, which is a derivate of the soil isolate mt-2 (11) and able to colonize the rhizosphere of several important crop plants (12).

BlastKOALA (13) analysis of the GM4FR genome revealed a gene encoding for a putative nematicidal protein (AidA) (14). Additionally, putative genes encoding insecticidal proteins such as *fitD*/*mcf* (K19615) and *tccC* (K11021) were identified. These insecticidal toxins are known from plant-associated *P. fluorescens* and *P. protegens* providing protective effects for their host plants (15–17). An antiSMASH 3.0.5 (18) analysis predicted two bacteriocin gene clusters, an arylpolyene gene cluster, and a nonribosomal polyketide synthetase (NRPS) cluster with no or low (<35%) similarity to known clusters. From the identified NRPS cluster, 9% of genes showed similarities to a pyoverdine gene cluster of *P. protegens* and *P. aeruginosa* (19). Pyoverdines are important virulence factors such as fluorescent siderophores and required in pathogenesis (20).

### Accession number(s).

This whole-genome shotgun project has been deposited at DDBJ/ENA/GenBank under the accession MKZO00000000. The version described here is version MKZO01000000.
